# Updated dataset for “Jellyfish in Hong Kong: a citizen science dataset”

**DOI:** 10.46471/gigabyte.196

**Published:** 2026-10-26

**Authors:** John Terenzini, Ashley Lau

**Affiliations:** ^1^ Hong Kong Jellyfish Project, Hong Kong, China; ^2^ Independent Researcher, Hong Kong, China

## Abstract

This work is an update and extension of the previously published article “Jellyfish in Hong Kong: a citizen science dataset”, by Terenzini et al. (2024). This Data Update consists of 516 jellyfish observations made by citizen scientists in Hong Kong during 2024 and 2025, who submitted photographs and videos to the Hong Kong Jellyfish Project website, iNaturalist project, and through social media. While citizen science is not new to China or, more specifically, Hong Kong, the Hong Kong Jellyfish Project is the first time citizen science has been used to investigate jellyfish in the region, filling the knowledge gap of a previously under-studied taxa in a biodiversity-rich region of the South China Sea. This Data Update has new species occurrence records for Hong Kong, including rarely seen species, taxonomically uncertain species, and updates previous records because of recent taxonomic revisions. This Data Update makes jellyfish data available for research into the roles and influences jellyfish have on Hong Kong’s highly impacted marine ecosystems. The potential usage of the Data Update extends to improving comprehensive marine management and tourism policies. This dataset is publicly available and discoverable in the Global Biodiversity Information Facility database.

## Data description

### Background

This work is an update and extension of the previously published article “Jellyfish in Hong Kong: a citizen science dataset”, by Terenzini et al. [1]. This Data Update describes an additional dataset of jellyfish observations from 2024–2025 submitted through the citizen science initiative Hong Kong Jellyfish Project’s (HKJP) website, iNaturalist project, and from social media (Facebook and Instagram). These observations were harmonized and deposited in the Global Biodiversity Information Facility (GBIF) repository.

Citizen science is a valuable tool for monitoring biodiversity, especially at a wider geographical and temporal scope than would be economically feasible for scientists using traditional methods [2]. Citizen science is not a new concept in China, where, for example, at the northern end of the South China Sea, marine citizen science projects have been used to examine seagrasses [3]. Jellyfish research using traditional methods, such as trawling, is extensive in the Yellow and Bohai seas of northern China [4], though to the knowledge of the authors, citizen science is not being used to investigate jellyfish elsewhere along the China coast.

Citizen science has been shown to be effective for uncovering jellyfish presence, seasonality, and distribution in countries around the Mediterranean [5]. The Hong Kong Jellyfish Project is a citizen science project examining jellyfish in Hong Kong’s local waters. HKJP research has described new species occurrence records [6], as well as described seasonality and distribution of jellyfish locally [7]. It increased knowledge of jellyfish distribution and transport throughout the South China Sea [8] and more broadly along the China coast [9]. The HKJP provides valuable information on an overlooked piece of Hong Kong’s extensive marine biodiversity [10]. Existing research in Hong Kong has only incidentally collected jellyfish data as part of a larger project [11] or have used jellyfish in laboratory settings [12].

### Context

In addition to the Context provided in “Jellyfish in Hong Kong: a citizen science dataset”, by [1], this Data Update is a timely addition to the known marine biodiversity of Hong Kong. With the recent adoption of the Kunming Montreal Global Biodiversity Framework (KMGBF) [13], member countries of the Convention on Biological Diversity are tasked with updating their national Biodiversity Strategy and Action Plan (BSAP). The central government of China requires Hong Kong, as a Special Administrative Region, to enact a city-level BSAP [14]. The KMGBF, and the Hong Kong BSAP (HKBSAP), recognizes the contributions that citizen science can make towards monitoring biodiversity and supports the inclusion of citizen science data for monitoring progress towards policy goals.

It is critical to understand what species are present and how their presence and distribution may be changing in order to examine their impacts on marine ecosystems and the ecosystem services these ecosystems provide [15]. Using citizen science data to fill the taxonomic data gaps in global biodiversity databases [16] is necessary for a more comprehensive understanding of marine biodiversity. Citizen scientists’ observations of jellyfish in [1] and this Data Update are an important contribution to the marine biodiversity of Hong Kong, as well as making this data available for use in marine policy. Jellyfish data generated by the Hong Kong Jellyfish Project has been included in the Biodiversity Information Hub, a requirement under the HKBSAP. The HKJP’s data is now available to use in marine policies addressing fisheries recovery and coastal management policies looking at impacts of jellyfish on recreational water users and Hong Kong’s tourism economy.

Additionally, because this dataset is available through GBIF in Darwin Core format, this dataset meets the principles for FAIR (Findable, Accessible, Interoperable, Reusable) [17] and is available for researchers globally. Other efforts to make jellyfish data globally available include the Jellyfish Database Initiative (JeDI), a database that collected four centuries of jellyfish data from published and unpublished records [18]. Data that is globally available increases its potential impacts by sharing with other researchers for use and allows for broader scope questions to be asked, such as the effects of climate change on marine biodiversity.

## Methods

There have been no changes to the Methods from the previous published paper [1].

### Study area

There have been no changes to the study area from the previous published paper [1].

### Sampling

There have been no changes to the sampling from the previous published paper [1].

### Data validation and quality control

Observations shared through the HKJP website, social media, or the iNaturalist project were first checked for quality control before identification. Observations that did not have photographs or videos of sufficient quality to be able to identify the subject were removed from the dataset. For example, a picture might be out-of-focus, too distant, or not contain a visible jellyfish. Other observations of jellyfish that were removed from the dataset came from captivity such as a school tank or Ocean Park, a local Hong Kong theme park with a jellyfish display. Observations that were repeated between both iNaturalist and the HKJP website were synthesized to a single observation. Locality information, either coordinates or a local place name or description, was collected from the citizen scientist observer on submission. In the case of a local place name, the map coordinates were entered into the dataset using the location given. Observations that passed the quality control procedures were identified to the lowest taxonomic level using literature sources ([19–21] and other species-specific literature) and consultation with experts, as needed.

This data update is available in Darwin Core format; 516 terms are available for the 2024–2025 dataset. All mandatory fields are present and have undergone validation and screening using the TaiBIF Integrated Publishing Toolkit (IPT) before uploading and publishing via the GigaScience Press GBIF page [22].

### Taxonomic scope

This Data Update records the presence of 38 jellyfish species in Hong Kong. Specifically, 15 scyphozoans (two to genus level), 15 hydrozoans (four to genus level), two cubozoans (one to genus level), and six ctenophores (three to genus level) (Table 1). Many of the species in this Data Update overlap with the species previously recorded in [1], however there are 9 new species records for Hong Kong included here (Table 1, indicated with “*”). One species record, *Physalia utriculus*, derives from a recent taxonomic change to the *Physalia* distributed around Hong Kong [23]. The genus *Physalia* was previously considered to consist only of *Physalia physalis*, until [23] used molecular and morphological methods to divide this genus into four species. Previous records of *Physalia physalis* in Hong Kong should be updated to *Physalis utriculus*. Another new species record, *Thysanostoma flagellatum*, is currently recognized as a separate species, but may be a variation on another previously recorded species, *Thysanostoma loriferum* [19]. These taxonomic changes and uncertainties highlight the continued need for more research into jellyfish species to help clarify species’ records. The remaining seven records are new species records reported for the first time in Hong Kong.

**Table 1:** Taxonomic scope of jellyfish identified to genus and species level for each class identified (in bold) for the Hong Kong Jellyfish Project. New species records for Hong Kong are indicated with “*”.

Order	Family	Genus	Species
**Scyphozoa**			
Rhizostomeae	Catostylidae	*Acromitus*	*Acromitus flagellatus*
		*Catostylus*	Catostylus townsendi*
	Rhizostomatidae	*Rhopilema*	*Rhopilema hispidum*
	Lobonemidae	*Lobonema*	*Lobonema smithii*
	Lychnorhizidae	*Anomalorhiza*	*Anomalorhiza shawi*
	Cepheidae	*Netrostoma*	
	Leptobrachidae	*Thysanostoma*	Thysanostoma flagellatum*
			*Thysanostoma loriferum*
	Mastigiidae	*Mastigias*	*Mastigias albipunctata*
		*Phyllorhiza*	*Phyllorhiza punctata*
Semaeostomeae	Cyaneidae	*Cyanea*	*Cyanea nozakii*
		*Cyanea*	*Cyanea purpurea*
	Pelagiidae	*Pelagia*	*Pelagia noctiluca*
		*Chrysaora*	*Chrysaora chinensis*
	Ulmaridae	*Aurelia*	
**Hydrozoa**			
Limnomedusae	Geryoniidae	*Liriope*	*Liriope tetraphylla*
Trachymedusae	Rhopalonematidae	*Aglaura*	*Aglaura hemistoma*
Anthoathecata	Corymorphidae	Euphysa*	
	Porpitidae	*Porpita*	*Porpita porpita*
		*Velella*	Velella velella*
	Pandeidae	*Amphinema*	
			Amphinema rubrum*
			Amphinema turrida*
Leptothecata	Aequoreidae	*Aequorea*	
			*Aequorea macrodactyla*
		Clytia*	
	Eirenidae	*Eugymnanthea*	Eugymnanthea japonica*
Siphonophorae	Agalmatidae		
	Physaliidae	*Physalia*	Physalia utriculus*
**Cubozoa**			
Carybdeida	Tripedaliidae	*Tripedalia*	*Tripedalia maipoensis*
	Carukiidae	*Morbakka*	
**Ctenophora**			
Beroida	Beroidae	*Beroe*	
			Beroe campana*
Lobata	Bolinidae	*Bolinopsis*	
			*Bolinopsis rubripunctata*
	Leucotheidae	*Leucothea*	
	Ocyropsidae	*Ocyropsis*	*Ocyropsis crystallina*

## Re-use potential

This Data Update improves knowledge of the jellyfish biodiversity of Hong Kong and can be used for research into the roles jellyfish play in local marine ecosystems and the impacts they have on those ecosystems. Knowledge of jellyfish populations and seasonality is essential for comprehensively integrating fisheries and conservation policies to effectively address jellyfish impacts [24]. For example, Hong Kong’s historically overfished waters have provided gaps for opportunistic species such as jellyfish to proliferate and potentially impact the recovery of fish communities after the 2012 trawling ban. The recovery of Hong Kong’s fish communities post-ban has been smaller than in other areas of the world where trawling bans have taken place [25] and jellyfish proliferation may be impeding fish community recovery through predation and competition, in conjunction with continued fishing pressures. Jellyfish monitoring data such as this Data Update, and data derived from a jellyfish monitoring network using citizen science in combination with traditional monitoring, would improve understanding of the impacts of jellyfish on Hong Kong’s sustainable development and use of its resources [15]. The new HKBSAP provides an opportunity for jellyfish monitoring to become a part of Hong Kong’s marine biodiversity monitoring to develop more effective marine management and tourism policies.

Beach and water-based tourism is increasing in Hong Kong, with over 7 million people visiting its beaches in 2023 [26], increasing the chances of encounters with jellyfish and subsequent impacts on marine tourism. The Hong Kong Tourism Board recommended promoting marine tourism to enhance the attractiveness of Hong Kong as a tourist destination [27]. The Hong Kong government stated that marine tourism crosses several policy areas and would have to be examined in regards to overall government policies [27]. This provides an opportunity for jellyfish data such as this Data Update and additional jellyfish monitoring data generated from an integrated monitoring network [15], to be used to examine the impacts of jellyfish on Hong Kong’s growing tourism industry.

## Data Availability

The dataset described here is hosted on the GigaScience Press GBIF page [22].

## Abbreviations

GBIF, Global Biodiversity Information Facility; HKJP, Hong Kong Jellyfish Project; IPT, Integrated Publishing Toolkit.

## Declarations

### Authors' information

JT is the founder and principal investigator of the Hong Kong Jellyfish Project.
