# Supplementary material for: Updated dataset for “Jellyfish in Hong Kong: a citizen science dataset”
Source: GigaByte. 2026 Oct 26;2026:gigabyte196. doi: 10.46471/gigabyte.196 (PMC13748680; doi:10.46471/gigabyte.196)
Supplement: Peer review history

# Reviewer report: Review MS


Reviewer name and names of any other individual's who aided in reviewer Ephrime B MetilloDo you understand and agree to our policy of having open and named reviews, and having your review included with the published papers. (If no, please inform the editor that you cannot review this manuscript.)YesIs the language of sufficient quality?YesPlease add additional comments on language quality to clarify if needed
Are all data available and do they match the descriptions in the paper? NoAdditional CommentsWhy were some specimens not identified to species level while others are? These genera with species are: Netrostoma, Aurelia, Euphysa, Cytia, and Morbakka,Are the data and metadata consistent with relevant minimum information or reporting standards? See GigaDB checklists for examples <a href="http://gigadb.org/site/guide" target="_blank">http://gigadb.org/site/guide</a>YesAdditional CommentsIs the data acquisition clear, complete and methodologically sound?YesAdditional CommentsIs there sufficient detail in the methods and data-processing steps to allow reproduction?YesAdditional CommentsIs there sufficient data validation and statistical analyses of data quality? NoAdditional CommentsUnclear. Authors should have used AI to run many images and videos to confirm species of jellyfish.Is the validation suitable for this type of data?YesAdditional CommentsIs there sufficient information for others to reuse this dataset or integrate it with other data?YesAdditional CommentsAny Additional Overall Comments to the AuthorI hope they can address my comments above.RecommendationMinor Revision


# Reviewer report: Review MS


Reviewer name and names of any other individual's who aided in reviewer Dan WANGDo you understand and agree to our policy of having open and named reviews, and having your review included with the published papers. (If no, please inform the editor that you cannot review this manuscript.)YesIs the language of sufficient quality?YesPlease add additional comments on language quality to clarify if needed
Are all data available and do they match the descriptions in the paper? YesAdditional CommentsAre the data and metadata consistent with relevant minimum information or reporting standards? See GigaDB checklists for examples <a href="http://gigadb.org/site/guide" target="_blank">http://gigadb.org/site/guide</a>YesAdditional CommentsIs the data acquisition clear, complete and methodologically sound?YesAdditional CommentsIs there sufficient detail in the methods and data-processing steps to allow reproduction?YesAdditional CommentsIs there sufficient data validation and statistical analyses of data quality? NoAdditional CommentsWhile I appreciate that the methodological framework aligns with the previous publication, data quality is not a static attribute that can be 'inherited' from a prior study. The newly added species represent a distinct batch of data acquisition. Therefore, the authors should provide independent summary statistics specifically for the new entries, rather than relying solely on a blanket statement of equivalence to the previous work.Is the validation suitable for this type of data?YesAdditional CommentsIs there sufficient information for others to reuse this dataset or integrate it with other data?YesAdditional CommentsAny Additional Overall Comments to the AuthorA significant weakness of the current abstract is the lack of a coherent scientific narrative for this updated version. The authors fail to establish three critical elements: (1) Background/Rationale – why is this extension necessary at this particular time (e.g., recent intensive field surveys, new taxonomic revisions)? (2) Highlights/Novelty – beyond the simple increase in species count, the abstract does not specify the unique features of the newly incorporated species (e.g., are they rare, cryptic, or geographically significant?). (3) Significance/Implications – the abstract does not articulate how this expanded dataset benefits the broader community. Does it fill a specific biogeographic gap, refine DNA barcoding references, or inform marine conservation policies? Without these elements, the abstract reads merely as a routine inventory rather than a valuable scientific contribution. A practical way to address this is to offer a concise summary of these three aspects (background, novelty, and significance) in the abstract, and defer the detailed exposition—including taxonomic justifications, quality control figures, and extended ecological context—to the relevant sections of the main text. This would keep the abstract informative yet readable, while ensuring the paper's core data and analyses are sufficiently detailed elsewhere.RecommendationMajor Revision


# Reviewer report: Re-Review MS


Indicate in the comments box below whether you are happy with the changes made or if the manuscript is unacceptable.Comments on revised manuscriptThe authors have adequately addressed my previous comments and made appropriate revisions to the manuscript. I am satisfied with the revised version and have no further comments. I support its acceptance for publication.Indicate in the comments box below whether you are happy with the changes made or if the manuscript is unacceptable.Comments on revised manuscriptThe authors have adequately addressed my previous comments and made appropriate revisions to the manuscript. I am satisfied with the revised version and have no further comments. I support its acceptance for publication.


# Editor report: Accept


Editor’s AssessmentThe manuscript is ready for formal acceptance.Editor’s AssessmentThe manuscript is ready for formal acceptance.
